# Supplementary material for: Cerebellar and hepatic alterations in ACBD5-deficient mice are associated with unexpected, distinct alterations in cellular lipid homeostasis
Source: Commun Biol. 2020 Nov 26;3:713. doi: 10.1038/s42003-020-01442-x (PMC7691522; doi:10.1038/s42003-020-01442-x)
Supplement: Supplementary file 1 — Supplementary Information [file 42003_2020_1442_MOESM1_ESM.pdf]

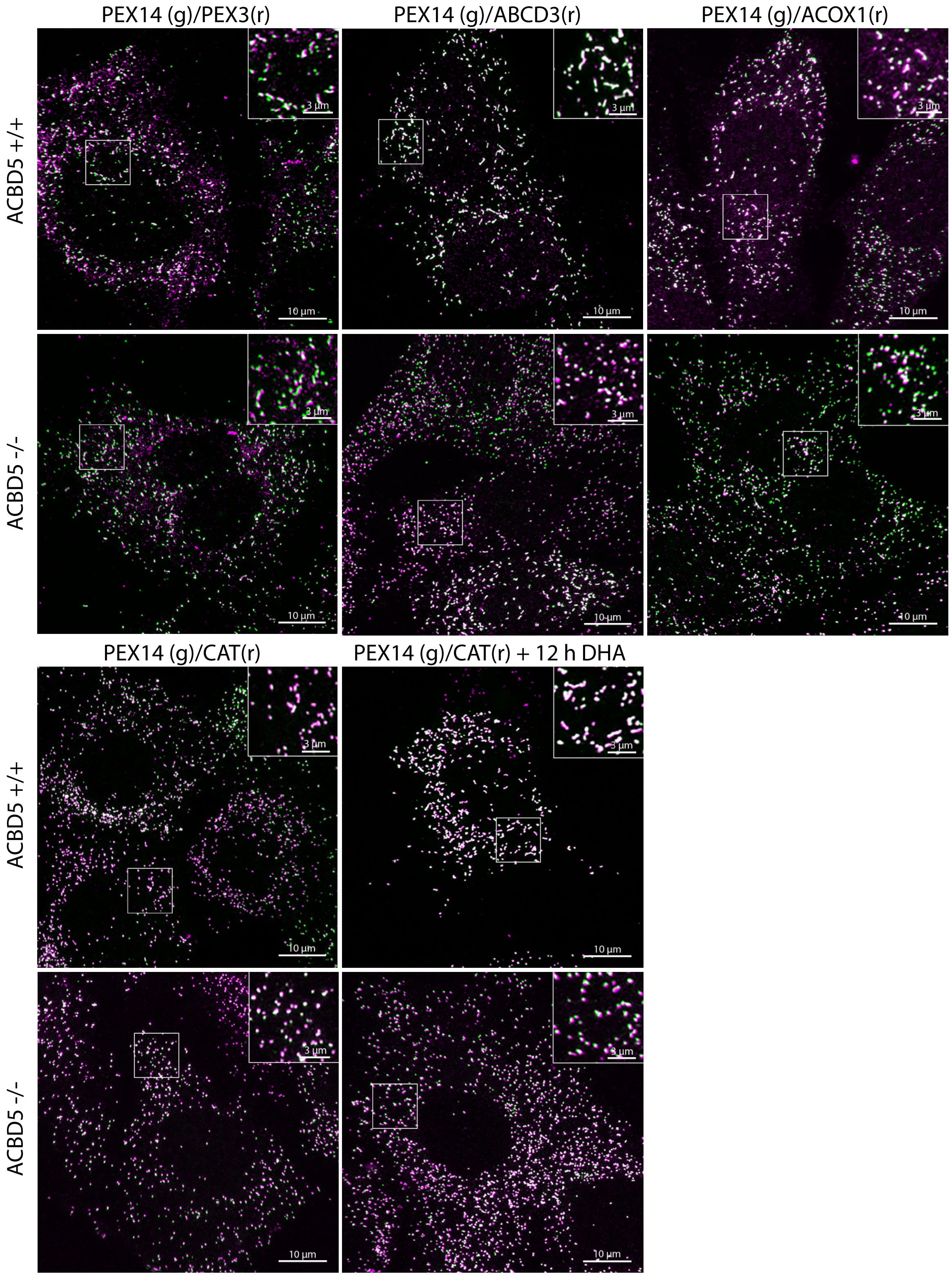

Supplementary Fig. 1: Distribution of peroxisomal proteins in *Acbd5*$^{+/+}$ and *Acbd5*$^{-/-}$ MEF.
In order to evaluate potential differences in peroxisome maintenance, MEF cultures were co-stained with antibodies against PEX14 (magenta) and PEX3, ABCD3, ACOX1 and catalase (green), respectively. All four proteins show a high degree of co-localization with PEX14 in both MEF lines. Note, that peroxisomes with a strong PEX14 signal can show low signal intensity for metabolic proteins (ABCD3, ACOX1) and vice versa, potentially indicating highly import competent and fully mature peroxisomes, respectively. No difference in the abundance and distribution of both peroxisome populations was obvious between *Acbd5*$^{+/+}$ and *Acbd5*$^{-/-}$ MEF. The PEX14 and catalase staining patterns after MEF incubation in 150 µm DHA highly colocalize in peroxisomes of various morphologies in both *Acbd5*$^{-/-}$ as well as *Acbd5*$^{+/+}$ MEF and did not reveal obvious differences in matrix protein import during the process of peroxisome maturation. Thus, PEX14 immunosignals were chosen as a representative marker for peroxisome elongation in quantitative measurements.

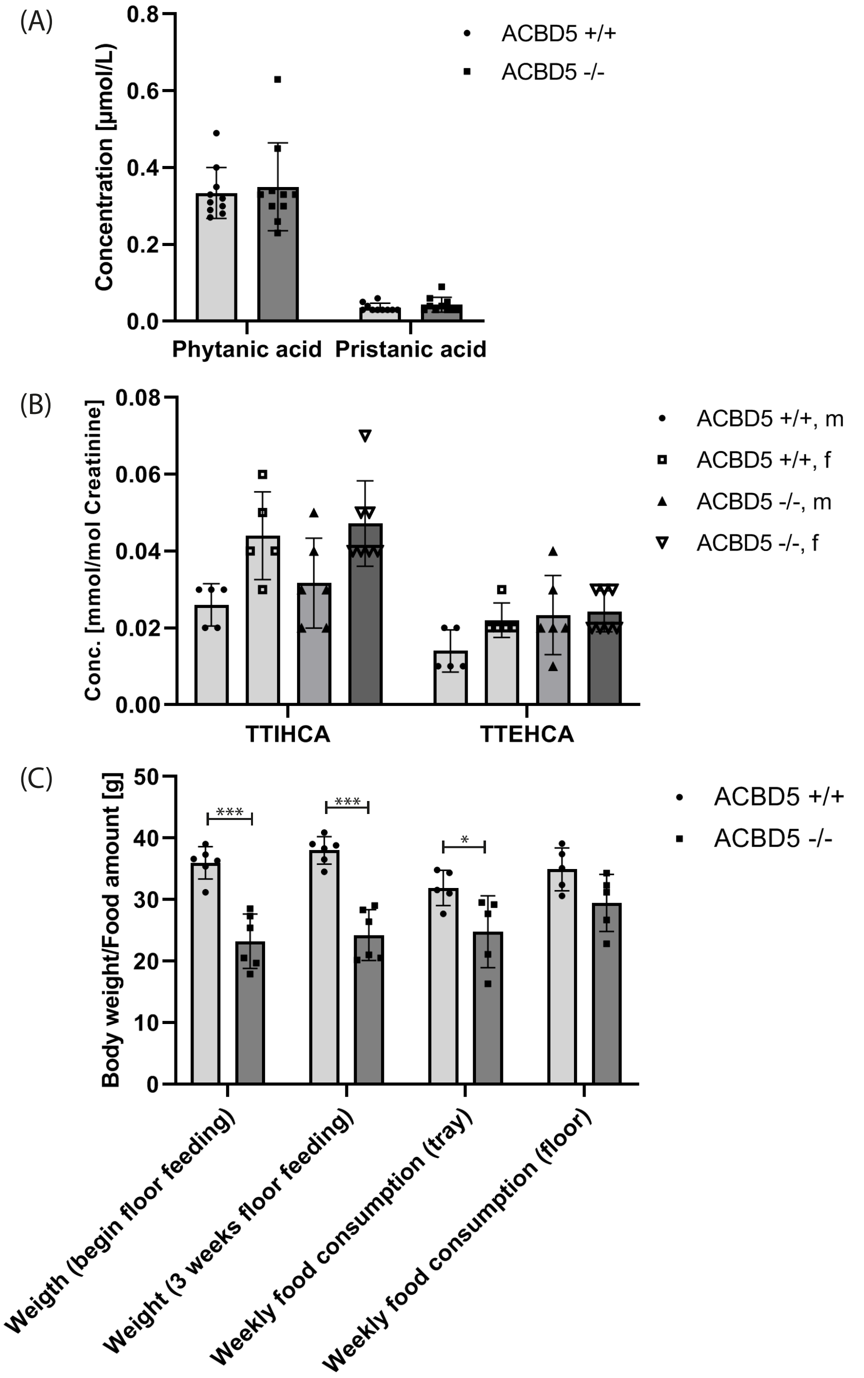

Supplementary Fig. 2: Bile acid concentrations and food consumption rates of *Acbd5*$^{-/-}$ mice
(A) Quantification of phytanic and pristanic acid in blood plasma of *Acbd5*$^{+/+}$ and *Acbd5*$^{-/-}$ mice (n=5 animals/gender and genotype). (B) Values for urinary diagnostic markers for a peroxisomal bile acid synthesis defect (Tauro-tri-hydroxycholestanoic acid – TTIHCA, Tauro-tetra-hydroxycholestanoic acid - TTEHCA) (n=5 for *Acbd5*$^{+/+}$ male and female mice, n=6 *Acbd5*$^{-/-}$ males, n=7 *Acbd5*$^{-/-}$ females). (C) Weight and food consumption rates of one year-old Acbd5$^{-/-}$ and *Acbd5*$^{+/+}$ mice (n=5 males/genotype). To analyze if the locomotor deficits observed in *Acbd5*$^{-/-}$ mice lead to reduced food uptake and body weight differences, male mice (n=5/genotype) were housed for three weeks in cages, in which food pellets were for easier accessibility placed on the cage floor instead into the tray at the cage top. Weight was determined before and at the end of the floor feeding period. When placed at the cage top *Acbd5*$^{-/-}$ in average consume significantly less food than *Acbd5*$^{+/+}$ mice. Food placement to the floor, however, did not raise food consumption rates to a significant extent and correspondingly no weight gain was observed for *Acbd5*$^{-/-}$ mice at the end of the experiment (all bar graphs show data means with standard deviations; *p<0.05, **p<0.01; unpaired t-test, two-sided).

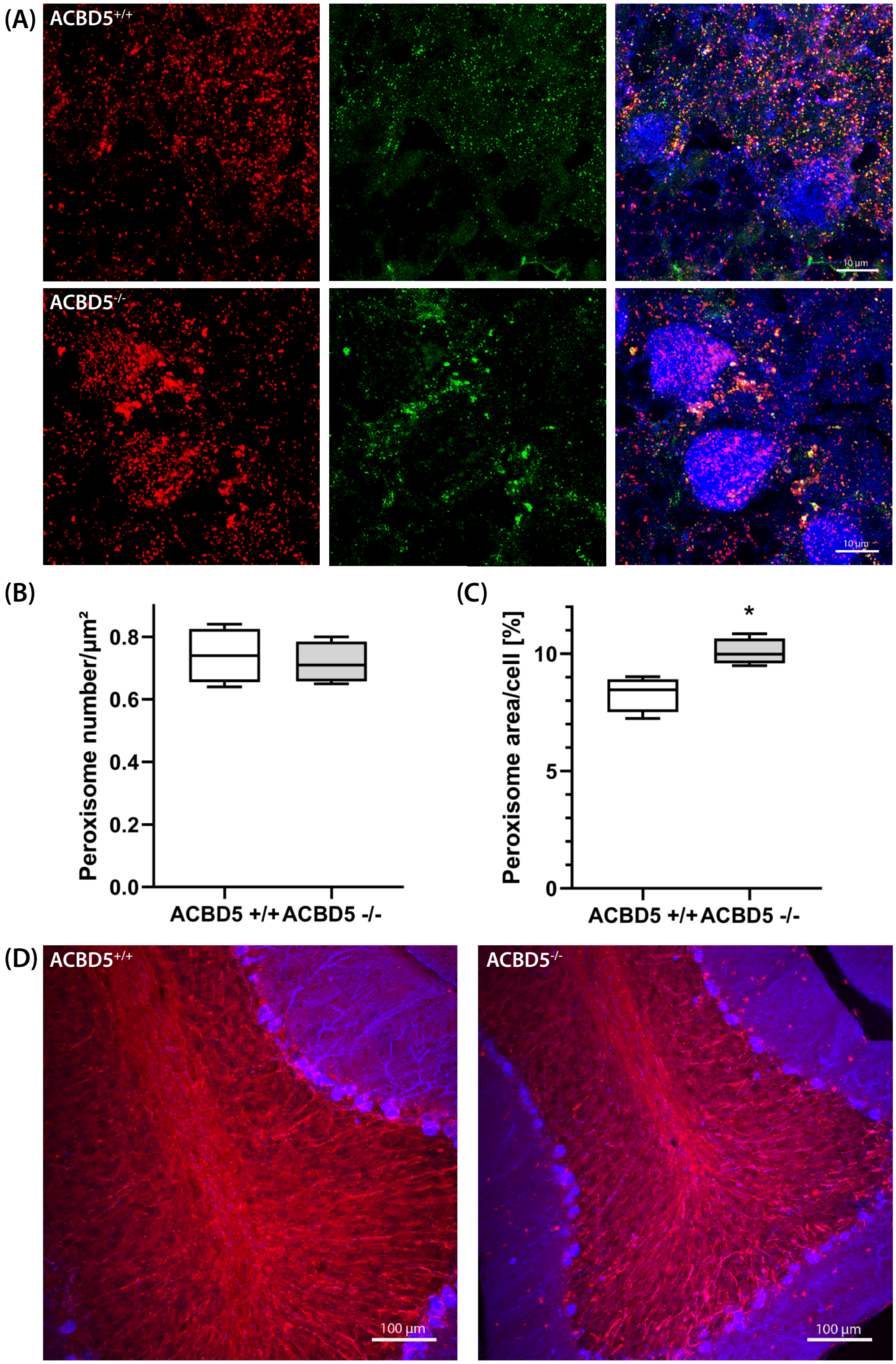

Supplementary Fig. 3: Peroxisome status and myelination in the cerebellum of *Acbd5*$^{-/-}$ mice.
(A) Peroxisomes stained with antibodies against PEX14 (red) and catalase (green), Purkinje cells were marked by calbindin antibodies (blue). Note, that Purkinje cells exhibit nearly no catalase signals, whereas a strong colocalization between catalase and PEX14 was obtained in adjacent regions in the Purkinje cell layer, most likely comprising peroxisomes in the soma of Bergmann glia. Images show maximum-projections of 5 serial z-planes of 0.25 µm. (B, C) Quantification of peroxisome abundance in Purkinje cells (single Z-plane in the nuclear plane) from *Acbd5*$^{+/+}$ and *Acbd5*$^{-/-}$ mice (n=4 mice/genotype, >25 Purkinje cells/mouse). While single particle counting revealed comparable numbers of peroxisomes/µm^2 (A), a slight difference (<10%) was obtained for the percentage area covered by PEX14 signals/*Acbd5*$^{-/-}$ compared to *Acbd5*$^{+/+}$ Purkinje cells (*p<0.05, **p<0.01; unpaired t-test, two-sided; box plots depict the interquartile range, medians and minima/maxima). (D) Immunofluorescence of myelin basic protein (MBP, red) as a marker for myelination of cerebellar fiber tracts. For better orientation calbindin (blue) was used to highlight Purkinje cells. In contrast to the immunoblotting experiments, no obvious differences in MBP staining intensities where observed. Immunofluorescence interassay variation among different cerebelli impeded a densitometry quantification of the MBP staining.

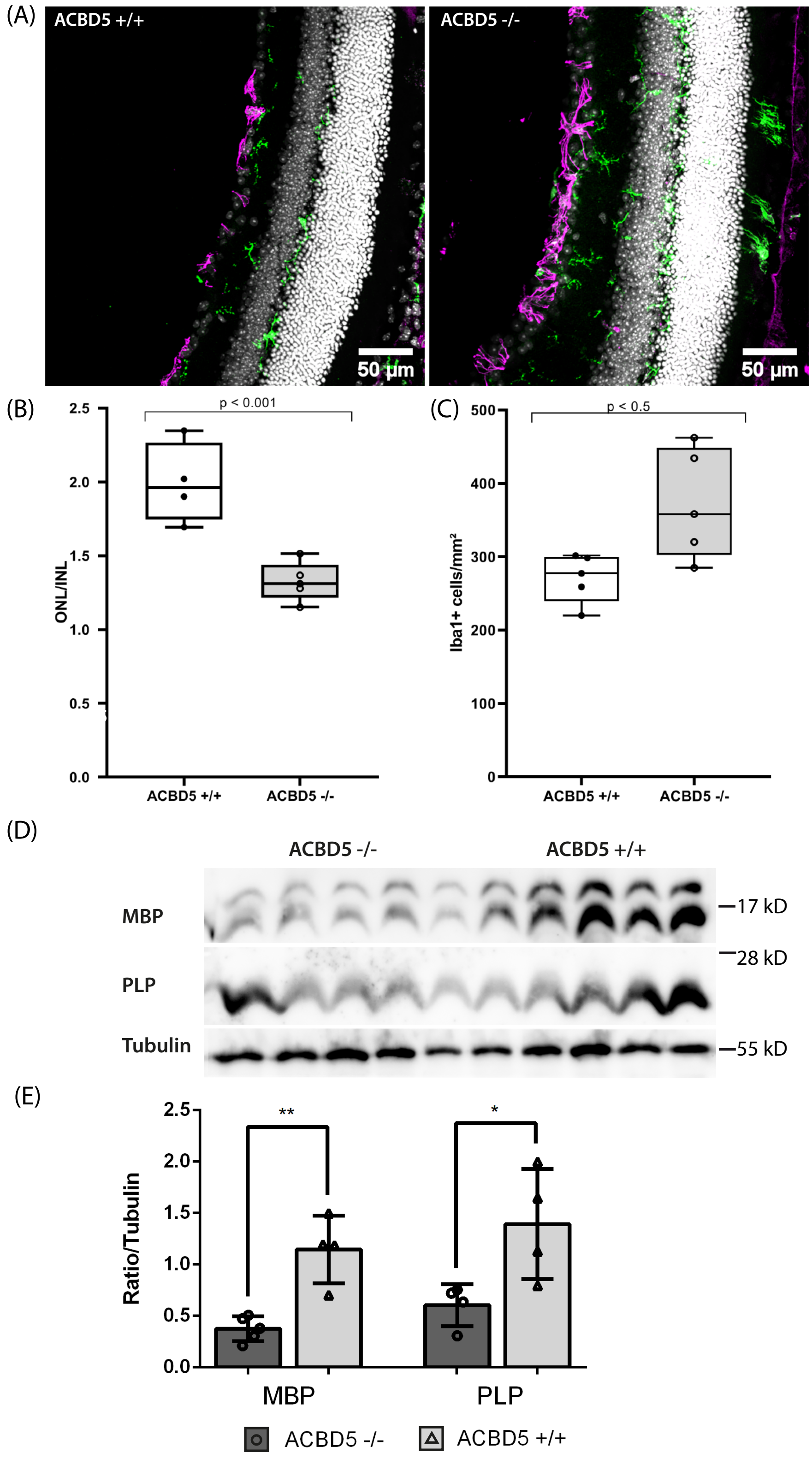

Supplementary Fig. 4: Retinal degeneration and cerebellar demyelination in 1 year-old *Acbd5*$^{-/-}$ mice
(A) Retina sagittal sections (n=5 mice/genotype) stained by IBA1 (microglia, green), GFAP (astroglia, magenta) and To-Pro-3 (nuclei, grey, Thermo-Fisher). (B) *Acbd5*$^{-/-}$ mice exhibit a significant reduction of the ratio between outer and inner nuclear layer thickness ($p<0.01$; unpaired, two-sided t-test) indicating a decline in the number of photoreceptor cells. (C) In parallel, numbers of microglial cells were found to be increased in *Acbd5*$^{-/-}$ retinas ($p<0.05$; unpaired t-test, two sided). Note also the striking invasion of activated microglia into the photoreceptor outer segment layer in *Acbd5*$^{-/-}$ mice and the stronger GFAP signal intensity in the stratum neurofibrarium indicating activation of astrocytes. (D) Immunoblots from an alternative set of cerebellar lysates against MBP and PLP to estimate axon myelinization in the cerebellum. Actin was applied as a loading control and used for normalization of the (E) MBP and PLP intensity quantification (*$p<0.05$, **$p<0.01$, ***$p<0.001$; unpaired t-test, two-sided). All bar graphs (B, C, E) show data means with standard deviations.

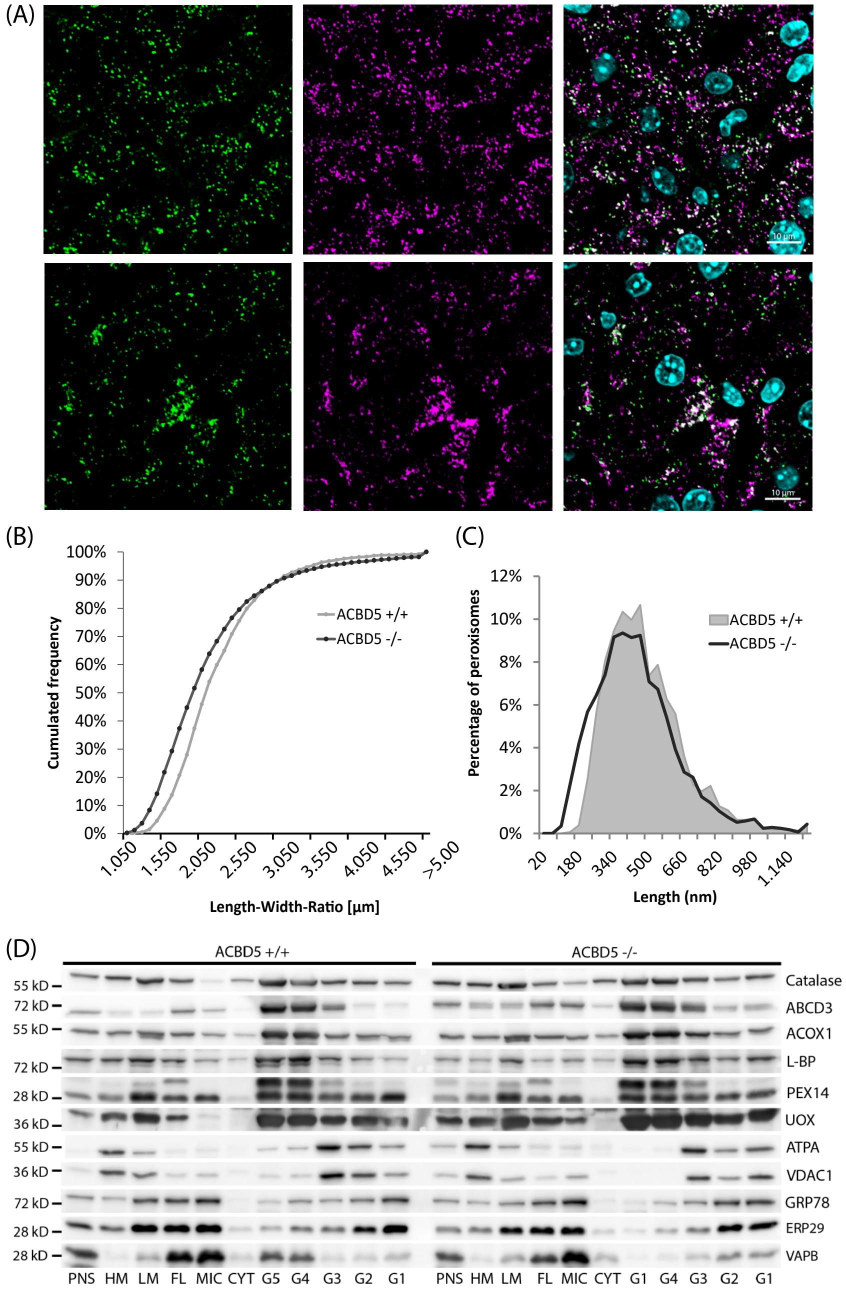

Supplementary Fig. 5: Supplementary information on peroxisomes in the liver of *Acbd5*$^{-/-}$ mice.
(A) Peroxisomes in liver tissue marked by catalase (green) and PEX14 (magenta) co-immunostaining, nuclei in the overlay were stained by To-Pro-3 (cyan, Thermo-Fisher). In both, *Acbd5*$^{-/-}$ as well as *Acbd5*$^{+/+}$ mice, peroxisome signals for PEX14 cover larger areas than for catalase, potentially indicating a large reservoir of nascent peroxisomes in the metabolically active hepatocytes. No significant differences in the ratio of catalase/PEX14 signals were obvious by microscopic inspection between both mouse strains. PEX14 signals cover a larger spectrum of the hepatocyte peroxisome population and were hence used for quantification of peroxisome abundance (see Fig. 5). (B) Length/width ratio of peroxisomes from hepatocytes of *Acbd5*$^{+/+}$ and *Acbd5*$^{-/-}$ mice as analysed by electron microscopy. (C) Size distribution of peroxisomes from hepatocytes of *Acbd5*$^{+/+}$ and *Acbd5*$^{-/-}$ mice observed by electron microscopy. Both measurements reveal tendency towards less elongated peroxisomes in *Acbd5*$^{-/-}$ mice (n= 1575 *Acbd5*$^{+/+}$ and 4613 *Acbd5*$^{-/-}$ peroxisomes). However, the predominant fraction of peroxisomes showed comparable size and morphology in both mice strains (size differences for the whole organelle population not significant). (D) Peroxisomal protein abundance in subcellular liver fractions of *Acbd5*$^{+/+}$ and *Acbd5*$^{-/-}$ mice from an alternative isolation series. Full size immunoblots are shown in Supplementary Fig. 10. Abbr: PNS – post nuclear supernatant, HM – heavy mitochondrial fraction, LM – light mitochondrial fraction, FL – fluffy LM layer, MIC – microsomal fraction, CYT – cytosol, G – gradient fractions (density increasing with numbers), L-BP – peroxisomal L-bifunctional protein, ATPA – ATP-synthase, subunit α. Columns G1 (low density) – G5 (high density) represent fractions from the final density gradient. Note that G5 and G4 comprise highly pure peroxisome fractions (see Islinger et al. 2007).

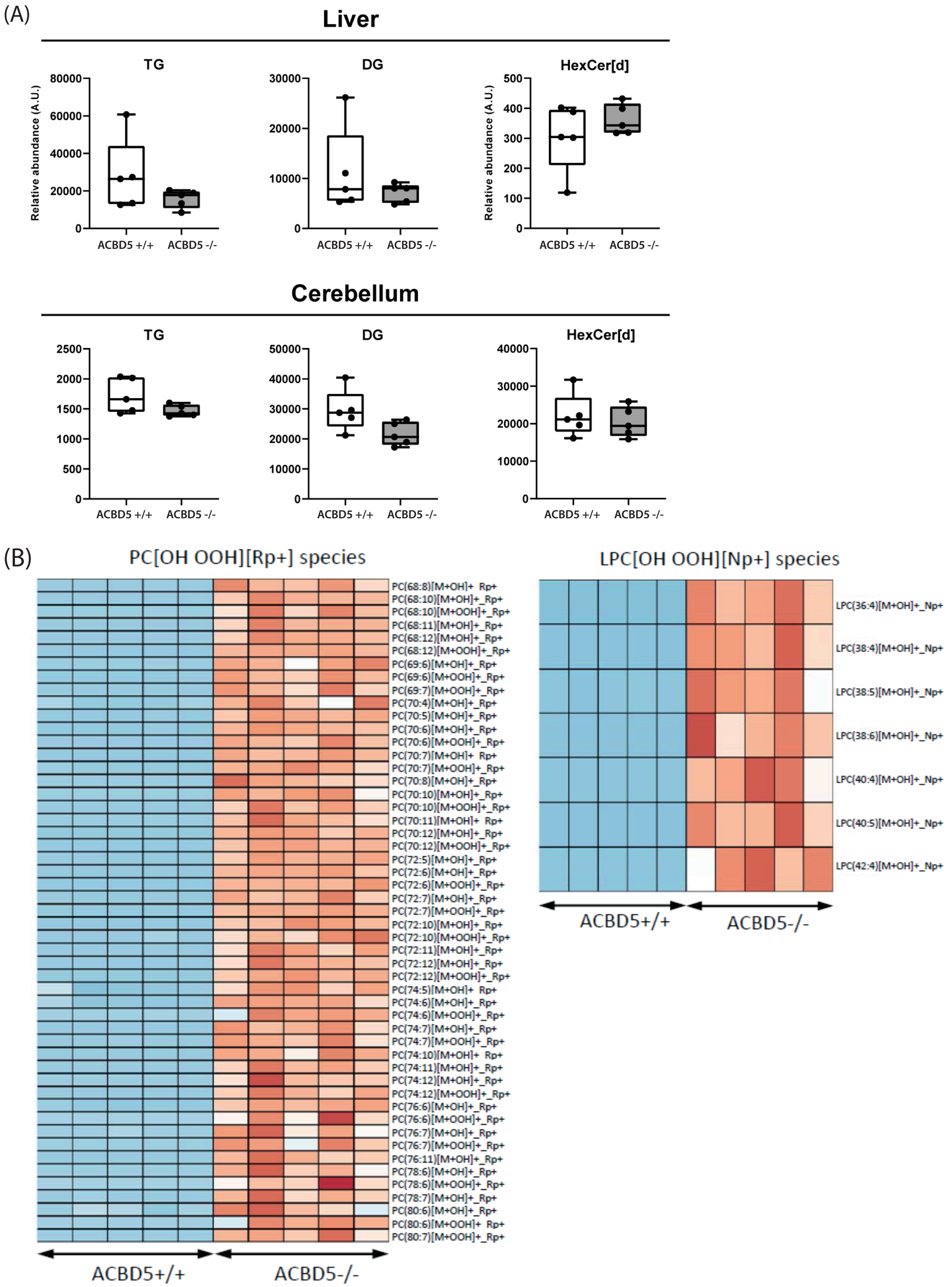

Supplementary Fig. 6: Supplementary results from the lipidome analysis.
(A) Levels of triacylglycerides, diacylglycerides and hexylceramides in liver and cerebellum of *Acbd5*$^{-/-}$ mice. Total lipid levels are defined as the summation of the relative abundance of all identified phospholipid species of the same class normalized to the corresponding internal standard, assuming identical response with respect to internal standard. The whisker blots depict the median, interquartile range and the maximums/minimums (*p<0.05, **p<0.01, ***p<0.001; unpaired t-test, two-sided; box plots depict the interquartile range, medians and minima/maxima). (B) Oxidized PC and LPC species accumulating in cerebelli *Acbd5*$^{-/-}$ mice; the top significantly altered lipid species are depicted by a colour gradient ranging from blue to red. For PC species results from Rp+ = reversed phase positive mode are shown; comparable values were found in PC[OH OOH][Np+] = normal phase positive mode (see source data to Fig. 7, 8). Based on LPC[OH OOH] composition and large m/z values of PC[OH OOH], accumulating species likely contain elongated PUFAs with 3 or 4 double bonds per fatty acid chain where one double bond has been oxidized (so originally 4 or 5 double bonds). A comparable accumulation of oxidized phospholipids with elongated PUFAs was neither observed for cerebellar PE species nor for PE and PC from liver.

MBP (Fig. 3)

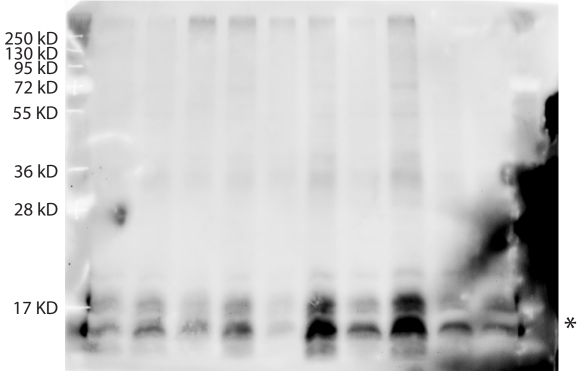

PLP (Fig. 3)

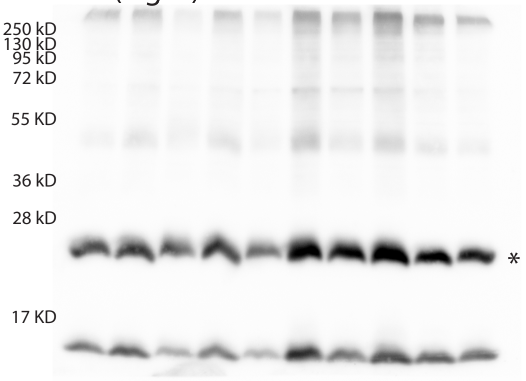

Actin (Fig. 3)

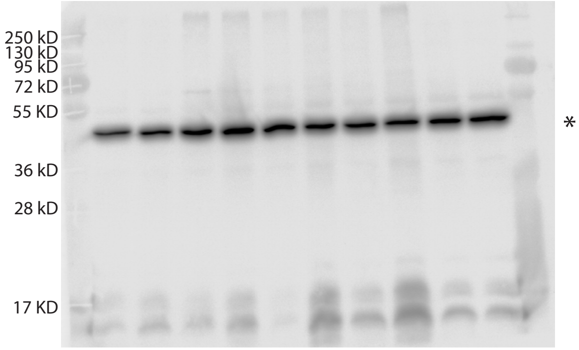

MBP (Suppl. Fig. 4)

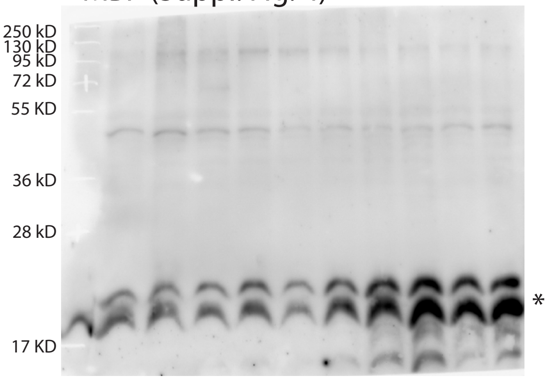

PLP (Suppl. Fig. 4)

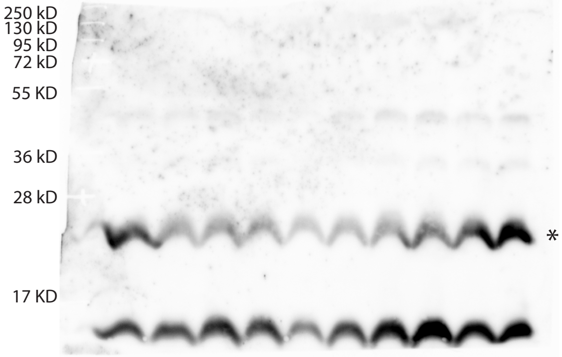

Tubulin (Suppl. Fig. 4)

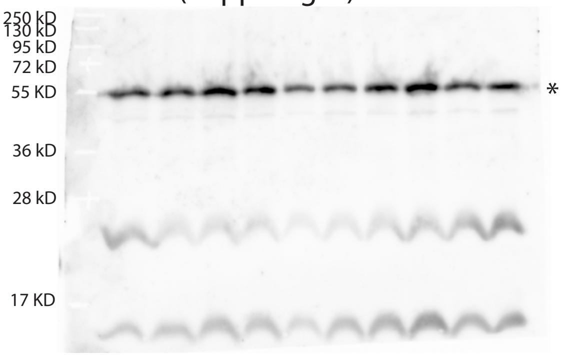

Supplementary Fig. 7: Uncropped images of the immunoblots from Fig. 3H and Supplementary Fig. 4D. The asterisks highlight the bands shown in Fig. 3H and Supplementary Fig. 4D.

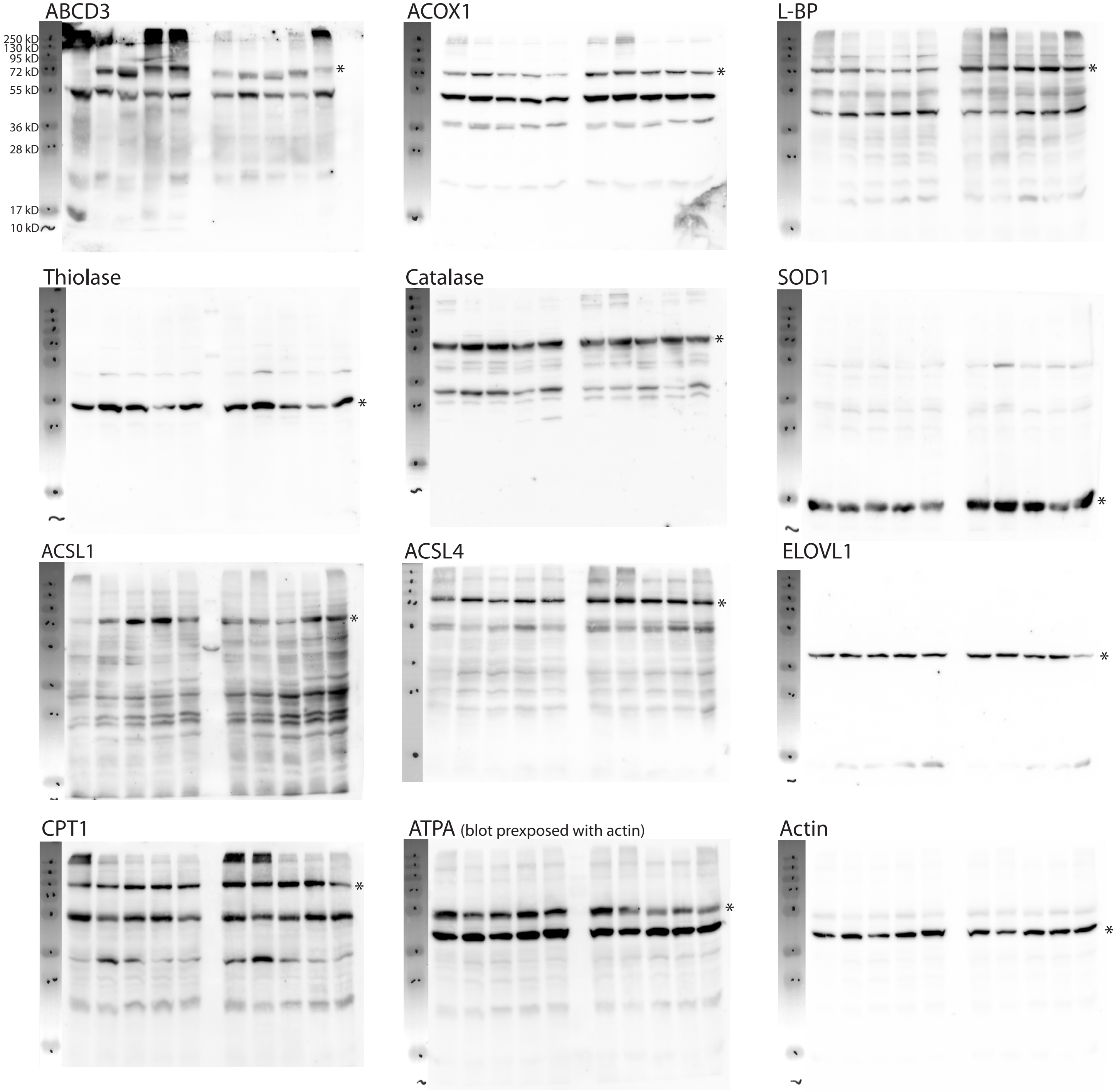

Supplementary Fig. 8: Uncropped images of the immunoblots from Fig. 6C.
The molecular weight (MW) standard shown at the left of each immunoblot was added from a separate image taken in parallel to monitor the prestained molecular markers. The MW standards shown were run in the middle lane of each gel. The asterisks highlight the bands shown in Fig. 6C.

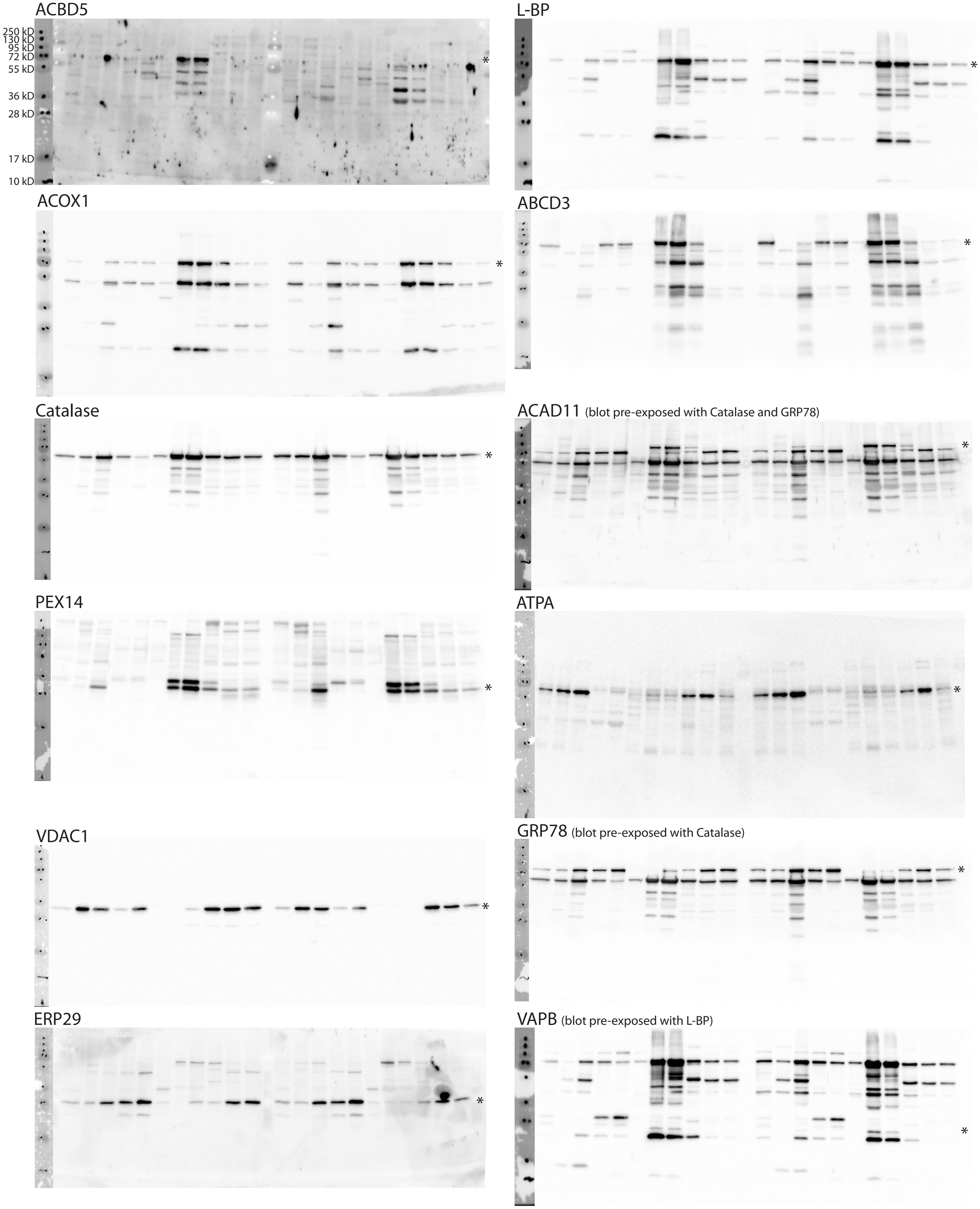

Supplementary Fig. 9: Uncropped images of the immunoblots from Fig. 6E.
The molecular weight (MW) standard shown at the left of each immunoblot was added from a separate image taken in parallel to monitor the prestained molecular markers. The MW standards shown were run in the middle lane of each gel. The asterisks highlight the bands shown in Fig. 6E.

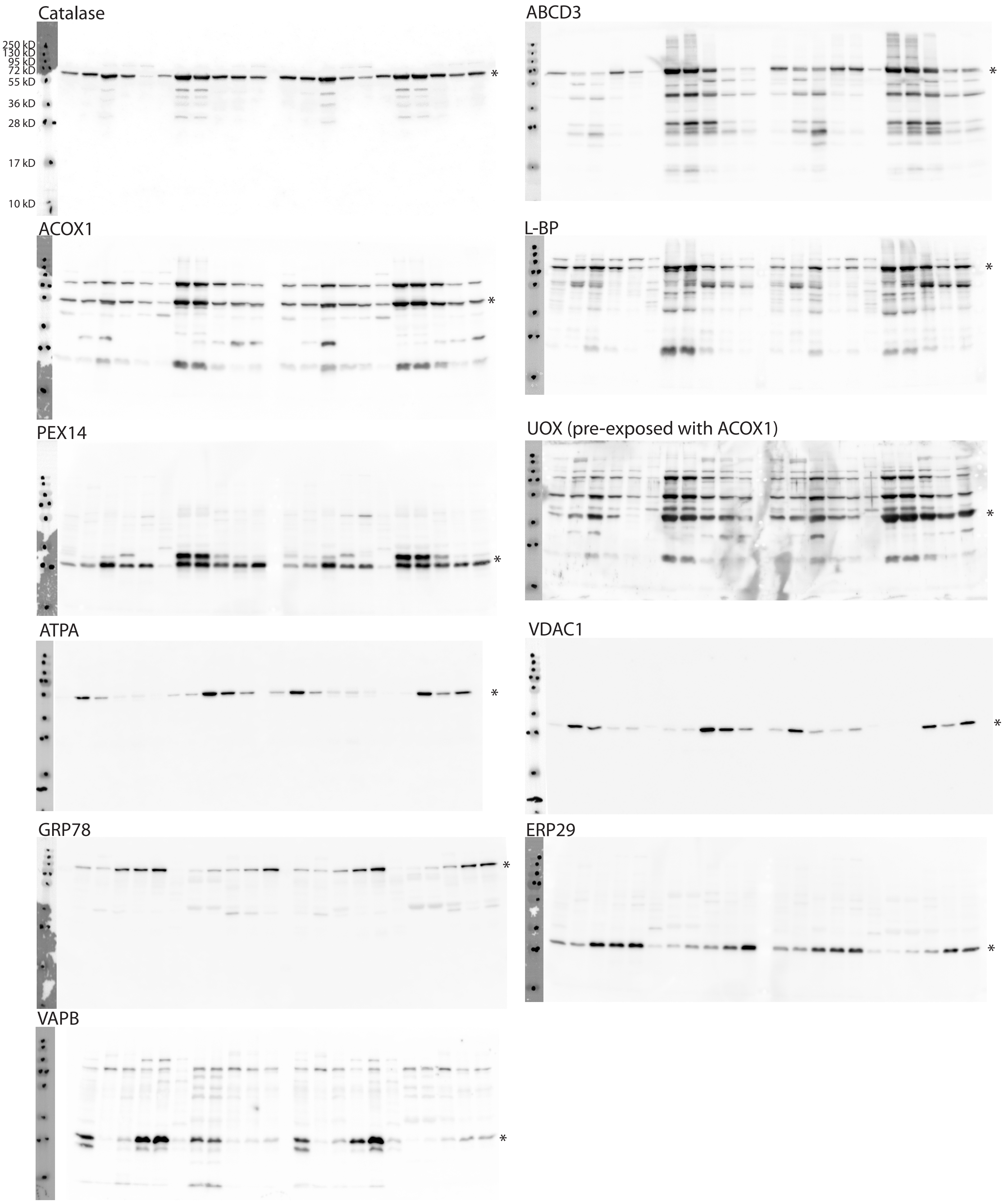

Supplementary Fig. 10: Uncropped images of the immunoblots from Supplementary Fig. 5D.
The molecular weight (MW) standard shown at the left of each immunoblot was added from a separate image taken in parallel to monitor the prestained molecular markers. The MW standards shown were run in the middle lane of each gel. The asterisks highlight the bands shown in Supplementary Fig. 5D.

Supplementary Table 1. Primers used in the study

Name	Sequence (5' to 3')
**ACBD5 genotyping**	
Acbd5_180388_For	TGAGTTCCTGCACACAAGTGG
Acbd5_180388_Rev	CAAGGGGAGGAGCATACCAG
CAS_R1_Term	TCGTGGTATCGTTATGCGCC
LacZ_2_For	ATCACGACGCGCTGTATC
LacZ_2_Rev	ACATCGGGCAAATAATATCG
**qPCR**	
ACBD5_ms_For	GGAGAGGGAGAGGGAATAGAAT
ACBD5_ms_Rev	TCTGTGAGGAGGGCTGATTA
ACBD4_PO_ms_For	TAGAGCCAGAGTTGGTGAGA
ACBD4_PO_ms_Rev	AACGTCCTTCATGCTTTCCT
GAPDH_ms_ For	TGGAAAGCTGTGGCGTGAT
GAPDH_ms_ Rev	TGCTTCACCACCTTCTTGAT

Supplementary Table 2. Primary and secondary antibodies used in this study

Primary Antibodies	Type	Dilution		Source
		IMF	WB	
ACOX1	rb		1:10000	Gift from T. Hashimoto, Shinshu University School of Medicine, Nagano, JP
ACOX1 (H00000051-B01P)	ms	1:100		Abnova Corporation, Taipei, Taiwan
ATPA (612516)	ms		1:20000	BD Transduction Laboratories,San Diego, USA
ACSL4 (sc-365478)	ms		1:1000	Santa Cruz Biotechonology, Santa Cruz, USA
PO-ketothiolase	rb		1:1000	Gift from A. Völkl, University of Heidelberg; Germany
ABCD3 (SAB4200181)	ms	1:200	1:1200	Sigma-Aldrich, Schnelldorf, Germany
Catalase	rb		10 µg/ml	Gift from A. Völkl , University of Heidelberg, Germany
Catalase (ab110292)	ms	1:200		Abcam, Cambridge, UK
ELOVL1 (orb 224117)	rb		1:1000	Biorbyt, Cambridge, UK
L-BP	rb		10 µg/ml	Gift from A. Völkl, University of Heidelberg, Germany
ACSL1 (13989-1-AP)	rb		1:2000	Proteintech, Rosemont IL, USA
CPT1 (sc-393070)	ms		1:1000	Santa Cruz Biotechonology, Santa Cruz, USA
SOD1 (ab13498)	rb		0.2 µg/ml	Abcam, Cambridge, UK
β-Actin (A5441)	ms		1:10000	Sigma, St Louis, USA
ELOVL1 (orb 224117)	rb		1:1000	Biorbyt, Cambridge, UK
ERP29 (ab11420-50)	rb		1:5000	Abcam, Cambridge, UK
VDAC1 (ab15895)	rb		1µg/ml	Abcam, Cambridge, UK
GFAP (173002)	rb	1:1000		Synaptic Systems, Göttingen, Germany
GRP78 (610979)	ms		1:1000	BD Transduction Laboratories San Diego, USA
ACAD11	rb		1:2000	Gift from G. Vockley, Pittsburg University, USA
PEX14 (custom made)	gp	1:2000		Proteogenix, Oberhausbergen, France
PEX14	rb		1:20000	Gift from D. Crane, Griffith University, Brisbane, Australia
PEX3	rb	1:200		Gift from G. Dodt, University of Tübingen, Germany
ACBD5 (ab100910)	rb	1:100	1:500	Abcam, Cambridge, UK
VAPB (HPA013144)	rb		1:2000	Sigma-Aldrich, Schnelldorf, Germany
ZO-1 (40-2300)	rb	1:100		Invitrogen Life Technologies, Eugene, USA
VGlut1 (135303)	rb	1:1000		Synaptic Systems, Göttingen, Germany
VGlut2 (135403)	rb	1:1000		Synaptic Systems, Göttingen, Germany
VGAT (131006)	ch	1:500		Synaptic Systems, Göttingen, Germany

MBP (ab7349)	rat	1:1000	1:15000	Abcam, Cambridge, UK
PLP (ab28486)	rb		1:1000	Abcam, Cambridge, UK
Ankyrin G (386005)	gp	1:1000		Synaptic Systems, Göttingen, Germany
Calbindin (214 004)	gp	1:1000		Synaptic Systems, Göttingen, Germany
Calbindin (300)	ms	1:1000		Swant, Marly, Switzerland
Calbindin (CB38)	rb	1:1000		Swant, Marly, Switzerland
IBA-1 (019 19741)	rb	1:2000		FujiFilm WAKO Chemical Corporation, JP
Alexa Fluor® 488 IgG	gt anti-gp	1:1000		Invitrogen Life Technologies, Eugene, USA
Alexa Fluor® 488 IgG	gt anti-rb	1:1000		Invitrogen Life Technologies, Eugene, USA
Alexa Fluor® 568 IgG	gt anti-rb	1:1000		Invitrogen Life Technologies, Eugene, USA
Alexa Fluor® 568 IgG	gt anti-gp	1:1000		Invitrogen Life Technologies, Eugene, USA
Alexa Fluor® 568 IgG	gt anti-rat	1:1000		Invitrogen Life Technologies, Eugene, USA
Alexa Fluor® 647 IgG	gt anti-ms	1:500		Invitrogen Life Technologies, Eugene, USA
Alexa Fluor® 647 IgG	gt anti-ch	1:500		Invitrogen Life Technologies, Eugene, USA
Alexa Fluor® 647 IgG	gt anti-gp	1:500		Invitrogen Life Technologies, Eugene, USA
HRP IgG (P0447)	gt anti-ms		1:5000	DAKO, Glostrup, Denmark
HRP IgG (P0448)	gt anti-rb		1:5000	DAKO, Glostrup, Denmark
