# Supplementary material for: Cerebellar and hepatic alterations in ACBD5-deficient mice are associated with unexpected, distinct alterations in cellular lipid homeostasis
Source: Commun Biol. 2020 Nov 26;3:713. doi: 10.1038/s42003-020-01442-x (PMC7691522; doi:10.1038/s42003-020-01442-x)
Supplement: Supplementary file 2 — Description of Additional Supplementary File [file 42003_2020_1442_MOESM2_ESM.docx]

Description of Additional Supplementary Files

Supplementary Data 1: Source data underlying plots shown in figures.

Supplementary Movie 1: Representative movie of a 1-year-old Acbd5+/+ mouse performing a ledge test. Note that the mouse is balancing on the edge of the cage wall without problems.

Supplementary Movie 2: Representative movie of a 1-year-old Acbd5-/- mouse performing a ledge test. Note that the mouse is even not able to stabilize itself on the edge of the cage wall. For clearer representation of the ataxic phenotype a mouse with a weak kyphosis was taken, which was rarely found at that age.
