# Supplementary material for: Cerebellar and hepatic alterations in ACBD5-deficient mice are associated with unexpected, distinct alterations in cellular lipid homeostasis
Source: Commun Biol. 2020 Nov 26;3:713. doi: 10.1038/s42003-020-01442-x (PMC7691522; doi:10.1038/s42003-020-01442-x)
Supplement: Supplementary file 7 — Reporting Summary [file 42003_2020_1442_MOESM7_ESM.pdf]

nature research

Corresponding author(s): M. Islinger, COMMSBIO-20-0556

Last updated by author(s): Oct 6, 2020

Reporting Summary

Nature Research wishes to improve the reproducibility of the work that we publish. This form provides structure for consistency and transparency in reporting. For further information on Nature Research policies, see Authors & Referees and the Editorial Policy Checklist .

Statistics

For all statistical analyses, confirm that the following items are present in the figure legend, table legend, main text, or Methods section.

n/a	Confirmed	
☐	☒	The exact sample size (*n*) for each experimental group/condition, given as a discrete number and unit of measurement
☐	☒	A statement on whether measurements were taken from distinct samples or whether the same sample was measured repeatedly
☐	☒	The statistical test(s) used AND whether they are one- or two-sided<br>*Only common tests should be described solely by name; describe more complex techniques in the Methods section.*
☒	☐	A description of all covariates tested
☐	☒	A description of any assumptions or corrections, such as tests of normality and adjustment for multiple comparisons
☐	☒	A full description of the statistical parameters including central tendency (e.g. means) or other basic estimates (e.g. regression coefficient) AND variation (e.g. standard deviation) or associated estimates of uncertainty (e.g. confidence intervals)
☒	☐	For null hypothesis testing, the test statistic (e.g. *F*, *t*, *r*) with confidence intervals, effect sizes, degrees of freedom and *P* value noted<br>*Give P values as exact values whenever suitable.*
☒	☐	For Bayesian analysis, information on the choice of priors and Markov chain Monte Carlo settings
☒	☐	For hierarchical and complex designs, identification of the appropriate level for tests and full reporting of outcomes
☒	☐	Estimates of effect sizes (e.g. Cohen's *d*, Pearson's *r*), indicating how they were calculated

Our web collection on statistics for biologists contains articles on many of the points above.

Software and code

Policy information about availability of computer code

Data collection	
Data analysis	Source code and binaries for the software used for the morphometric AIS analysis are available on github (https://github.com/jhnnsrs/aisuite2)<br>Source codes and binaries used for the S3EM 3D-reconstructions will be given by the authors upon any request.

For manuscripts utilizing custom algorithms or software that are central to the research but not yet described in published literature, software must be made available to editors/reviewers. We strongly encourage code deposition in a community repository (e.g. GitHub). See the Nature Research guidelines for submitting code & software for further information.

Data

Policy information about availability of data

All manuscripts must include a data availability statement. This statement should provide the following information, where applicable:
- Accession codes, unique identifiers, or web links for publicly available datasets
- A list of figures that have associated raw data
- A description of any restrictions on data availability

The authors confirm that the data supporting the findings of this study are available within the article [and/or] its supplementary material. Upon reasonable request, raw mass spectrometry data of the lipidomics analysis can be made available.

Field-specific reporting

Please select the one below that is the best fit for your research. If you are not sure, read the appropriate sections before making your selection.

Life sciences study design

All studies must disclose on these points even when the disclosure is negative.

Sample size	As no data on the expectable magnitude of alterations in the ACBD5-/- mouse model was available, samples sizes were calculated on presumptions to obtain a α-error of < 0.05% and a β-error of < 20%.
Data exclusions	No data was excluded from the analyses.
Replication	Experiments were repeated for three times and produced reproducible results
Randomization	The study is based on the comparative analysis of inbred mouse strains. Age- and gender-matched animals were picked at random in all experiments.
Blinding	In case of behavioral tests and microscopy image acquisition investigators were blinded to group allocation.

Reporting for specific materials, systems and methods

We require information from authors about some types of materials, experimental systems and methods used in many studies. Here, indicate whether each material, system or method listed is relevant to your study. If you are not sure if a list item applies to your research, read the appropriate section before selecting a response.

Materials & experimental systems

n/a	Involved in the study	
☐	☒	Antibodies
☐	☒	Eukaryotic cell lines
☒	☐	Palaeontology
☐	☒	Animals and other organisms
☒	☐	Human research participants
☒	☐	Clinical data

Methods

n/a	Involved in the study	
☒	☐	ChIP-seq
☒	☐	Flow cytometry
☒	☐	MRI-based neuroimaging

Antibodies

Antibodies used

All data on antibodies used in the study are described in Table S2

Validation

All primary antibodies used for Immunoblotting were validated by (1) validation of the correct MW of the detected bands and (b) by organelle-specific signal-enrichment in subcellular fractions from mice and rats, which were previously intensively characterized by enzymatic activity profiling and proteome analysis (Islinger et al. 2007, JBC 282, 23055-23069; Islinger et al. 2010, J. Proteome Res. 9, 113-124).

All primary antibodies used for immunofluorescence microscopy were validated (a) by omitting primary antibody incubation in one of the sections on the each slide; (b) the gp Pex14 antibody by colocalization with the intensively characterized rb Pex14 antibody produced by D. Crane, Brisbane (Grant P, et al. 2013, Histochem Cell Biol 140, 423-442); (C) by information accessible at the manufacturer website:

GFAP (173002), Synaptic Systems, https://www.sysy.com/products/gfap/facts-173002.php

vGlut1 (135303), Synaptic Systems, https://www.sysy.com/products/vglut1/facts-135303.php

vGlut2 (135403), Synaptic Systems, https://www.sysy.com/products/vglut2/facts-135403.php

vGAT (131006), Synaptic Systems, https://www.sysy.com/products/vgat/facts-131006.php

Ankyrin G (386005), Synaptic Systems, https://www.sysy.com/products/ankyrin/facts-386005.php

Calbindin (214004), Synaptic Systems, https://www.sysy.com/products/calbindin/facts-214004.php

Calbindin (300), Swant, https://www.swant.com/?p=products&c=1.1

Calbindin (CB38), Swant, https://www.swant.com/?p=products&c=1.1

IBA-1 (019 19741), FujiFilm Wako, https://fujifilmcdi.com/products-services/icell-products/anti-iba/?gclid=EAIaIQobChMIvu6n-4SS6AIVBeN3Ch2BvQrfEAAYASAAEgJB8PD_BwE

ACBD5 (ab100910), Abcam, https://www.abcam.com/acbd5-antibody-ab100910.html

MBP (ab7349), Abcam, https://www.abcam.com/myelin-basic-protein-antibody-12-ab7349.html

ZO-1 (40-2300), Invitrogen, https://www.thermofisher.com/antibody/product/ZO-1-Antibody-clone-ZMD-437-Polyclonal/40-2300

ACOX1 (H00000051-B01P), Abnova Corporation, http://www.abnova.com/products/products_detail.asp?catalog_id=H00000051-B01P

Catalase (ab110292), Abcam, https://www.abcam.com/catalase-antibody-12c2db9-peroxisome-marker-ab110292.html

Eukaryotic cell lines

Policy information about cell lines

Cell line source(s)	Mouse embryonic fibroblasts (MEF) from the mouse strains specified bellow were prepared as described in the manuscript's "Materials and Methods" section
Authentication	For MEF validation immunofluorescence microscopy using fibroblast-specific anitbodies was performed. As shown in Fig. 1, both MEFs were stained for the presence of ACBD5.
Mycoplasma contamination	The MEFs were tested for mycoplasma contamination giving negative results
Commonly misidentified lines (See ICLAC register)	**N/A**

Animals and other organisms

Policy information about studies involving animals; ARRIVE guidelines recommended for reporting animal research

Laboratory animals	Mouse strains used in the study (C57BL/6N-Atm1Brd Acbd5tm1a(EUCOMM)Wtsi/WtsiCnbc, C57BL/6N) are described described in the section "Materials and Methods". Ages and gender of the mice used in individual experiments are specified in the manuscript text.
Wild animals	The study did not involve wild animals
Field-collected samples	The study did not involve samples collected from the field
Ethics oversight	Animal breeding and experiments were approved by the Regierungspräsidium Karlsruhe under 35-9185.81/G-202/19

Note that full information on the approval of the study protocol must also be provided in the manuscript.
